# Supplementary material for: Insights into the Cell Division of Neospora caninum
Source: Microorganisms. 2023 Dec 28;12(1):61. doi: 10.3390/microorganisms12010061 (PMC10818811; doi:10.3390/microorganisms12010061)

Supplementary Table S1. Centrosomal proteins of *Toxoplasma gondii* and *Plasmodium spp.* that have homology in *N. caninum*.

Gene Name	*Neospora caninum* (NCLIV_)	*T. gondii* Gene ID (TGME49_)	*P. falciparum* Gene ID (Pf3D7)
CEP72	33520	233940	1347800
CEP110	11540	211430	1032800
CEP164	57420	314358	-
CEP120	14980	285210	-
CEP76	46370	226610	-
POC1	59430	216880	826700
SAS6	44630	306430	607600
Sfi1	33740	274000	-
CEP135	23900	-	626500
Centrin 1	63750	247230	107000
Centrin 2	66190	250340	1446600
Centrin 3	26390	260670	1027700
Centrin 4	51230	237490	1105500
CEP170	22960	201790	1307800
CEP250	44680	212880	-
kif24	13810	287160	1245100
CEP 530	* Ncaninum_LIV_000303500	246190	-
Nek2/NimA	43340	292140	1228300
CEP250L1	* Ncaninum_LIV_000499800.1	290620	-
PP1	54750	310700	1414400
CEP131	20060	205590	-
LLRC45	4080	209830	-
SAS6L	11960	301420	1316400
SAS-4/C-PAP	27800	258710	1458500

* Centrosomal proteins of *Toxoplasma gondii* and *Plasmodium* spp. that have homology in *Neospora caninum.* Note that the majority of the centrosomal proteins are the same in *Toxoplasma gondii* and *Neospora caninum.* Table adapted from [14].

Supplementary Figure S1. A 3D computational model from UExM unveils the intricacies of *N. caninum* vacuoles. Pv: parasitophorous vacuole. Host cell nuclei are indicated.

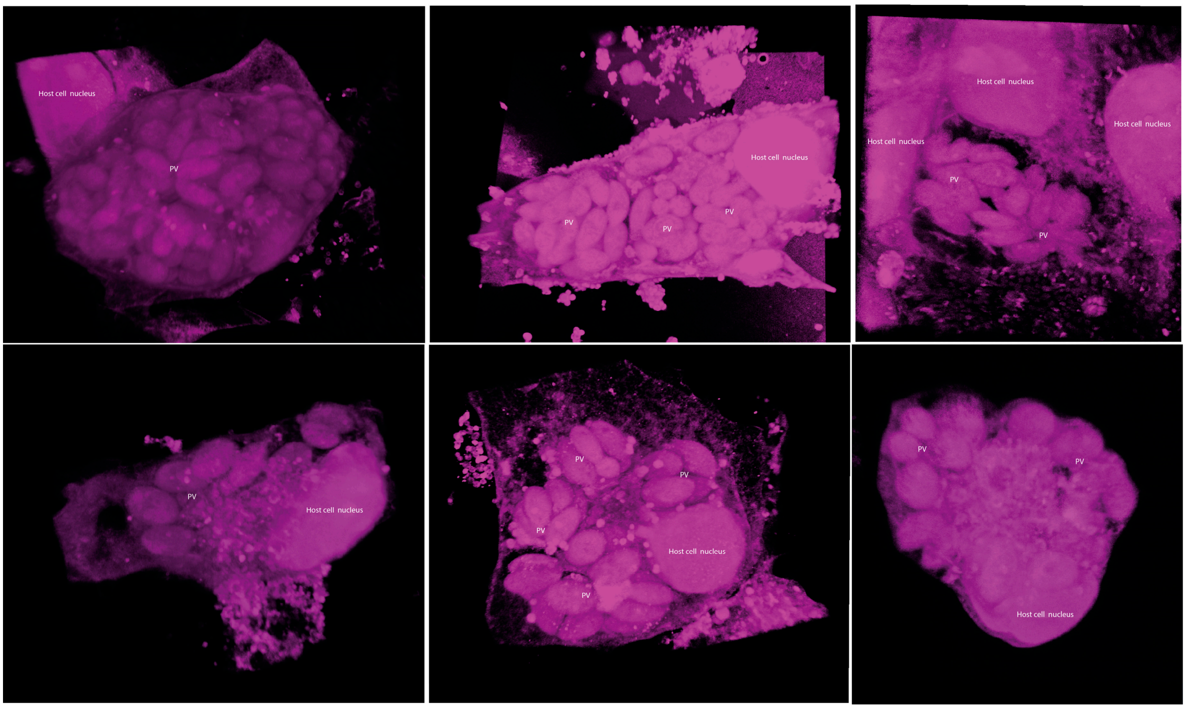

Supplementary Figure S2. Subpellicular microtubules in *N. caninum*. All the observed tachyzoites showed a 22-microtubule arrangement.

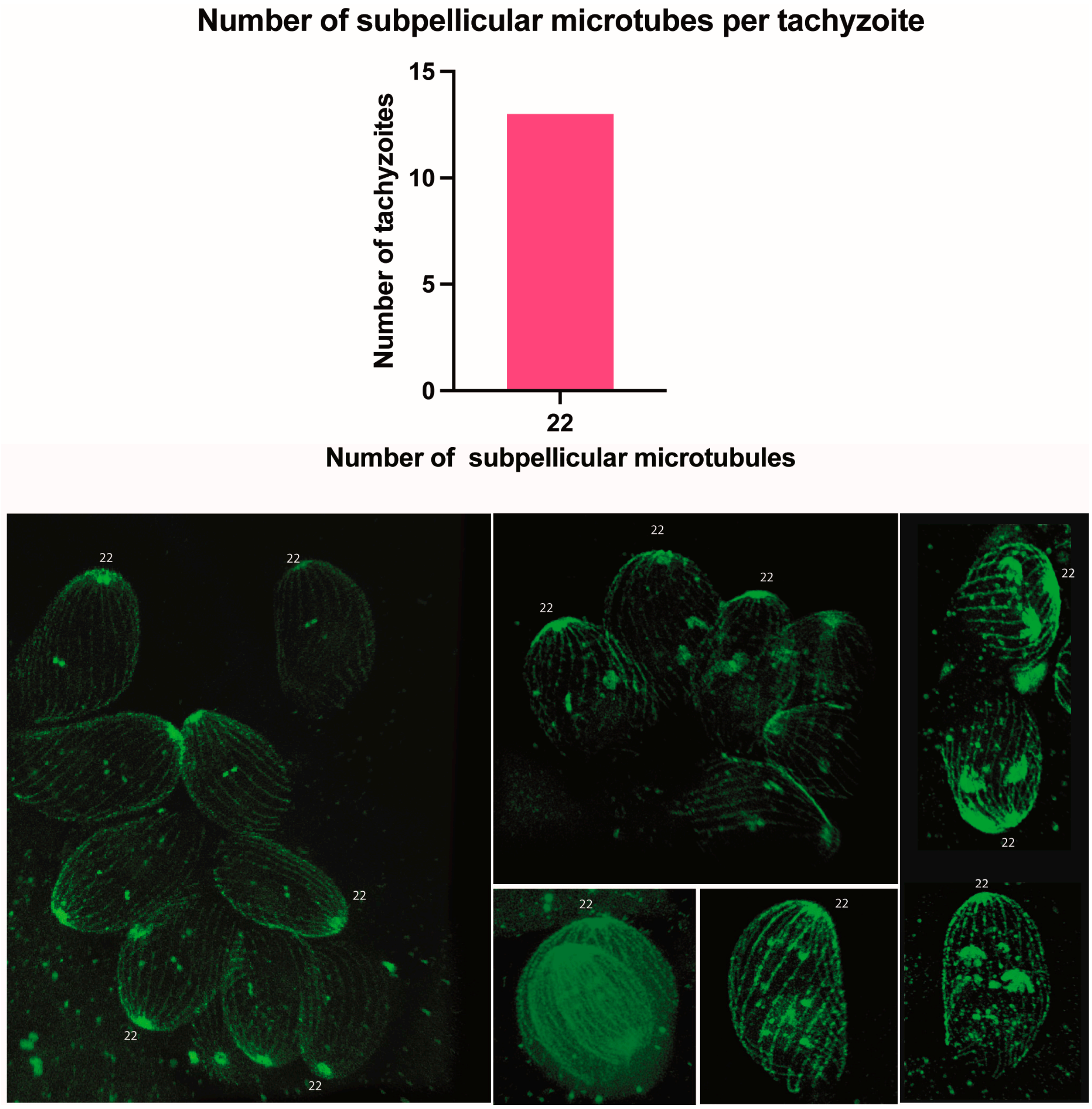

Supplement: Supplementary file 1 [file microorganisms-12-00061-s001.zip › microorganisms-2724205-supplementary.pdf]
